# Prevalence of non-cardiac incidental findings during routine clinical CMR assessment

**DOI:** 10.1186/1532-429X-11-S1-P48

**Published:** 2009-01-28

**Authors:** Thomas R Burchell, Didier Locca, Anthony Mathur, Ceri Davies, Mark A Westwood

**Affiliations:** London Chest Hospital, UK, London, UK

**Keywords:** Hiatus Hernia, Adult Congenital Heart Disease, Large Hiatus Hernia, Cross Sectional Imaging Modality, Black Blood Image

## Introduction

Over the last few years there has been a substantial increase in the use of cross sectional imaging modalities to assess the heart. Cardiac MRI (CMR) is now routinely used in the assessment of a variety of cardiac pathologies. It is not uncommon to identify non-cardiac abnormalities during image review. The frequency of non-cardiac findings has been previously addressed using cardiac CT, with 7 published studies totalling 2,495 patients. The prevalence of non-cardiac findings was 529 (21%) with a range of 5% to 69%. The majority of the abnormalities were pulmonary, most commonly small isolated nodules (<10 mm). The diversity and significance of these incidental findings has to date not been assessed with CMR.

## Objectives

The purpose of our study was to assess the prevalence of incidental non-cardiac findings found during routine CMR assessment.

## Methods

The reports of 714 consecutive adult CMR studies, performed for all current clinical indications with the exception of adult congenital heart disease, were reviewed retrospectively. These were performed between February and August 2008 on a Philips Achieva CV 1.5 Tesla MRI scanner (Philips Medical Systems, Best, The Netherlands). The studies were performed and reported by consultant cardiologists, in accordance with SCMR standardised acquisition protocols.

A multislice single shot steady state sequence was used as an initial scout scan. Three orthogonal planes were acquired (transverse, coronal and sagittal) with a large field-of-view coverage. Axial black blood images were also obtained (10 to 16 slices) using turbo spin-echo and a double inversion prepulse (TE/TR = 20/1500 ms).

## Results

The mean patient age was 61.3 +/- 15 years (range 16 to 86 years), and 476 (67%) were male. 218 (24%) of the patients had previously undiagnosed non-cardiac abnormalities. These included, renal cysts (9%), aortic pathology (3%), lung collapse/consolidation (2%), liver cysts (2%), lung mass (1%). There were also 2 cases of mediastinal masses, 1 mediastinal lympahadenopathy, 6 increased signal from the hila, 1 renal mass and one large hiatus hernia.

## Conclusion

The detection rate of non-cardiac findings on routine CMR scans is relatively high and broadly similar to that of cardiac CT. Unlike CT, CMR detects very few incidental lung findings. The CMR scout scan has a larger field of view than cardiac CT, allowing routine imaging of the abdomen, which explains the high number of intra-abdominal abnormalities.

Further work is needed to assess the clinical significance of these findings.

